# Broadband Near-Infrared Absorber Based on All Metallic Metasurface

**DOI:** 10.3390/ma12213568

**Published:** 2019-10-30

**Authors:** Ke Zhang, Ruixiang Deng, Lixin Song, Tao Zhang

**Affiliations:** 1 Key Laboratory of Inorganic Coating Materials CAS, Shanghai Institute of Ceramics, Chinese Academy of Sciences, Shanghai 200050, China; zhangke@student.sic.ac.cn (K.Z.); dengrx@mail.sic.ac.cn (R.D.); 2 Center of Materials Science and Optoelectronics Engineering, University of Chinese Academy of Sciences, Beijing 100049, China

**Keywords:** metamaterial, broadband absorber, near-infrared, localized surface plasmon resonance

## Abstract

Perfect broadband absorbers have increasingly been considered as important components for controllable thermal emission, energy harvesting, modulators, etc. However, perfect absorbers which can operate over a wide optical regime is still a big challenge to achieve. Here, we propose and numerically investigate a perfect broadband near-infrared absorber based on periodic array of four isosceles trapezoid prism (FITP) unit cell made of titanium (Ti) over a continuous silver film. The structure operates with low quality (Q) factor of the localized surface plasmon resonance (LSPR) because of the intrinsic high loss, which is the foundation of the broadband absorption. The high absorption of metal nanostructures mainly comes from the power loss caused by the continuous electron transition excited by the incident light inside the metal, and the resistance loss depends on the enhanced localized electric field caused by the FITP structure. Under normal incidence, the simulated absorption is over 90% in the spectrum ranging from 895 nm to 2269 nm. The absorber is polarization-independent at normal incidence, and has more than 80% high absorption persisting up to the incident angle of ~45° at TM polarization.

## 1. Introduction

In 2008, Landy et al. [1] first experimentally demonstrated the perfect metamaterial absorber, which works in microwave frequency band. Since then, many works concentrate on metamaterial absorber operating at frequency from microwave range [2,3] through terahertz [4,5], infrared [6,7], and into the visible region [8,9,10]. In the optical region, perfect metamaterial absorber has been an important branch of optical devices for its unique performance. It has undergone a rapid and flourishing development, finding a wide variety of potential application including thermal emitters [6,11], bolometers [12], solar energy harvesting [8,13], photodetections [7], and sensing [9,14,15]. The typical structure of metamaterial absorber is metal-dielectric-metal (MDM) structure [6,7,16], consisting of patterned subwavelength periodic metal arrays on top of the central dielectric layer, backed by a metallic ground plane. Beyond that, some absorbers composed of plasmonic nanostructure/nanoparticles [14,17,18] and metasurfaces [19,20,21,22,23,24,25,26] structure have been proposed and studied extensively. The absorption enhancement of incident light is derived from various types of electromagnetic (EM) resonances, such as propagating surface plasmon resonance (PSPR) [6], localized surface plasmon resonance (LSPR) [23], surface lattice resonance (SLR) [19,26], and so on. The perfect absorption is usually a single or multiple narrow band because of their nature of the resonances. However, the perfect broadband absorption is highly required for applications in the fields of energy harvesting and thermal emission.

To achieve a large absorption bandwidth there are two common and direct strategies [27]. One method is planar arrangement [28,29], which requires to create multiple resonances in each unit cell via tailoring the size of the patterns on the top layer. In this case, multiple resonances [8,30] will appear and perfect absorption can be achieved by incorporating resonators into a unit cell. Specifically, if these resonance absorption peaks are close enough, they will overlap with each other to form broadband absorption. The second method is vertical arrangement [31,32,33], which can lead to broadband absorption by stacking multiple top layers in the vertical direction. One can use different dielectrics and metals as an individual bilayer and stack these bilayers to form a consecutive broadband absorption spectrum by tuning the geometric parameters simultaneously. However, for the method one, the number of the resonators is restricted because of the competition of neighboring resonances, and the absorption performance is sensitive to the structure size, thus its absorption intensity and bandwidth expansion are limited. For method two, the broadband absorption performance can be improved further by increasing the number of metal/dielectric layers. Nonetheless, it is rather difficult to realize experimentally since they require precise control in complex fabrication processes, which also limit their potential applications. Therefore, finding a simple and effective strategy to achieve perfect broadband absorber is a challenging and significant task.

Metasurfaces are two dimensional arrays formed by subwavelength periodic nanostructures of metal and dielectric. This structure can easily localize the incident light and enhance absorption because of its effect of localized surface plasmon resonance. However, the existing researches [10,19,21,23,26] mainly use noble metal or dielectric materials as the metasurface to achieve high absorption, which is difficult to achieve broadband absorption. High loss materials can excite low-Q surface plasmon resonances (SPRs), which is conducive to broadband absorption [34,35]. Therefore, we consider using high loss materials to design appropriate metasurface structure, aiming to widen the absorption peaks and get the perfect broadband absorber.

In this article, we propose a broadband and polarization-independent absorber based on simple and all metallic metasurface in near-infrared region. The structure consists of metallic array made of four isosceles trapezoid prism (FITP) unit cell over a continuous metal film. The absorption mechanism is explored through the analysis of the electric field distributions and the power loss profiles. Moreover, the absorption performance is numerically researched when the materials of the ground plane and the nanostructures are replaced by the other metal materials, respectively. For absorption spectrum, the effect of the geometric parameters of the unit cell structure on the absorption bandwidth is studied. Additionally, numerical simulations show the properties of the polarization angle and incident angle.

## 2. Structure Design and Simulations

**Figure 1:**
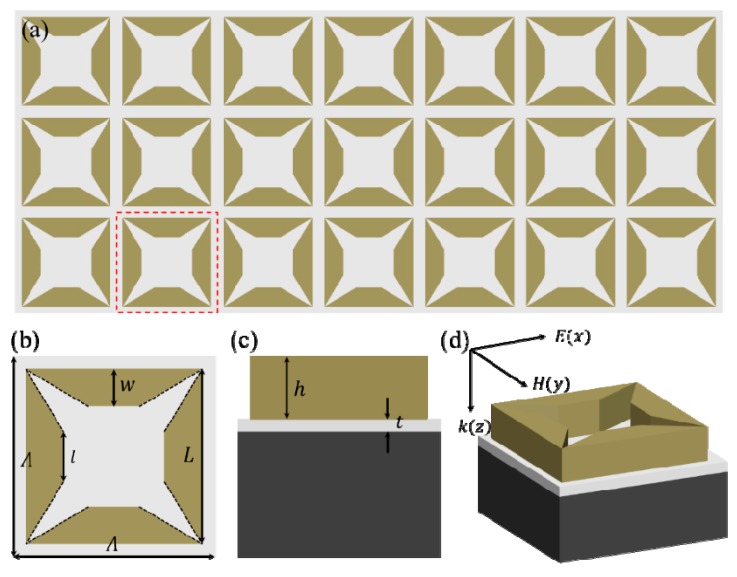
Schematic illustration of the proposed broadband absorber: (**a**) two-dimensional (2D) nanostructure array; (**b**–**d**) front, lattice, and perspective views of the unit cell nanostructure.

As shown in Figure 1, the proposed broadband absorber is composed of metallic array made of FITP unit cell backed with a continuous metallic film on a silicon substrate. The optimized geometric parameters of the broadband absorber are: *Λ* = 800 nm, *L* = 700 nm, *w* = 150 nm, *h* = 280 nm, *t* = 100 nm. It should be noticed that the metallic FITP structure is deformed from a metallic square ring structure. It is expected that the FITP structure can give rise to stronger resonance mode so as to result in a continuous strong broadband absorption in near-infrared region [36].

To investigate its absorption properties and mechanisms of the proposed absorber, full wave simulation was performed by a finite-different time-domain (FDTD) method based on commercial software (FDTD Solutions, Lumerical, Canada). In simulation, the bottom metallic layer is selected as silver (Ag) [37] film, the thickness of which is 100 nm, much larger than the typical skin depth in near-infrared regime. Since the incident light cannot go through Ag film, the absorption can be calculated by *A*(*λ*) = 1 − *R*(*λ*), where *R*(*λ*) is the reflection as functions of wavelength *λ*. Titanium (Ti) [37] is used to composed the metasurface because of its priority in features of high loss and stability in optical region. In addition, we will explore the effect of using other different metal materials (such as gold (Au), aluminum (Al), chromium (Cr), nickel (Ni), tungsten (W)) [37] as metallic ground plane and nanostructures on the absorption performance of the absorber in Section 3. The complex dielectric constant of all metals is fitted by a Drude-Lorentz model (with 6 coefficients) in the FDTD solutions. In simulation process, periodic boundary conditions were performed on unit cell in the x and y plane to represent the behavior of an infinite array at normal incidence. The structure is illuminated by an incident light with electric field parallel to the x-axis. For oblique angles, Bloch boundary conditions were performed on unit cell in the x and y plane. In both cases, a perfect matching layer was used in the z direction.

## 3. Results and Discussions

**Figure 2:**
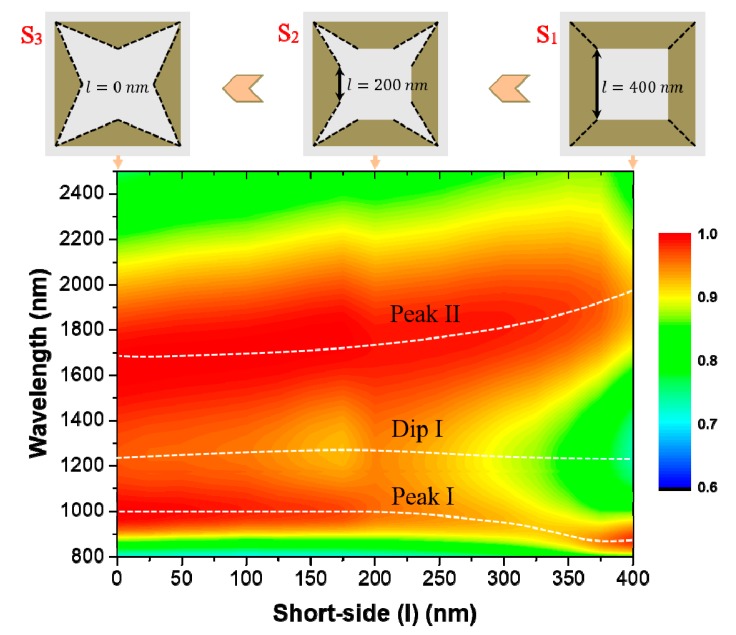
The simulated 2D absorption (*A(λ)*) color map as functions of the short-side length (*l*) and the wavelength. Parameters: *Λ* = 800 nm, *L* = 700 nm, *w* = 150 nm, *h* = 280 nm, *t* = 100 nm.

As mentioned above, the metallic FITP structure is deformed from a metallic square ring structure. To be specific, we can split the square ring into four isosceles trapezoids and get the proposed structure by decreasing the length of the short-side (*l*). Figure 2 shows the simulated 2D absorption color map with the short-side decreasing from 400 nm to 0 nm. We can see that these structures have similar absorbing behavior with two peaks and one dip (the white dotted line in Figure 2). The absorption performance of the square ring structure is poor because its absorption spectrum has a deeper dip. However, when just decreasing the length of the short-side, the absorption performance of the structure becomes better. For quantitative comparison of absorption performance, the relative bandwidth of the absorber [6], defined as *RBW* (≥90%) = 2 × (*λ_l_* − *λ_s_*)/(*λ_l_* + *λ_s_*), where *λ_l_* and *λ_s_* are the long and short limits of a wavelength range with absorption above 90%, respectively. Therefore, we can find that the *RBW* (≥90%) first increases and then decreases as the short-side length decreases. When the short-side length is smaller than 275 nm, the *RBW* (≥90%) of absorbers can reach above 80%. Furthermore, the designed absorber has best absorption performance which is greater than 90% from 895 nm to 2269 nm and the *RBW* (≥90%) reaches as high as 86.9%, when the short-side length is 275 nm.

**Figure 3:**
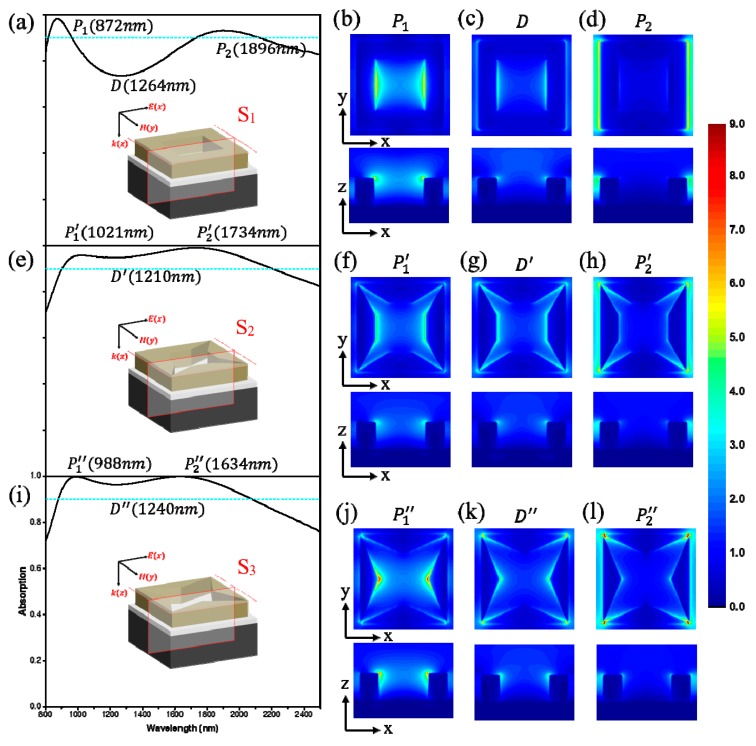
Absorption spectrum and electric field intensity distributions of the three structures at normal incidence. (**a**,**e**,**i**) Absorption spectrum of the structure S_1_, S_2_, and S_3_. (**b**–**d**) The electric field distributions of S_1_ at *P*_1_, *D*, and *P*_2_. (**f**–**h**) The electric field distributions of S_2_ at *P*_1_’, *D’*, and *P*_2_’. (**j**–**l**) The electric field distributions of S_3_ at *P*_1_″, *D″*, and *P*_2_″.

To reveal the working mechanism behind the stronger broadband absorption, we comparatively studied the electric field distributions of the three structures S_1_, S_2_, and S_3_ (the plane structure is illustrated in Figure 2, corresponding length of short-side are 400 nm, 200 nm and 0 nm, respectively) at different absorption peaks and dips. Figure 3b–d shows the electric field distributions of the structure S_1_. For peak *P*_1_ the localized electric fields are concentrated at the inner edge while it locates at the outer edge for peak *P*_2_. Obviously, the two absorption peaks originate from the excitation of the LSPR [6,23,26]. Since Ti is dispersive and has a relatively large imaginary part, the intrinsic absorption coefficient of Ti is very big, resulting in a rather low Q-factor for broadened absorption bandwidth [6,38]. However, at the dip *D*, the electric field is distributed at both sides of the square ring, and the electric field intensity is lower than that of the peaks, which leads to a poor absorption.

When the length of the short-side is decreased to 200 nm, the square ring structure becomes the four isosceles trapezoid structure (S_2_). As shown in Figure 3e, the intensity of the dip and the two peaks all exceeds 90%, indicating better absorption property of the structure S_2_. According to Figure 3f–h, the localized electric field mainly concentrates on the short-side at the peak *P*_1_’. It suggests that the absorption intensity at *P*_1_’ will be lower than that at *P*_1_, agreeing with the absorption spectrum. Different from the structure S_1_, E-field of structure S_2_ is distributed at the corner and the inner side at the dip *D’*, while the E-field is mainly localized in the corner at the peak *P*_2_’. The absorption intensity at dip *D’* and peak *P*_2_’ both increase because of a stronger localized electric resonance result from smaller length of the short-side.

Finally, when the length of short-side is decreased to zero, we can get the structure S_3_. As shown in Figure 3i, broadband and high absorption is achieved, but the bandwidth is slightly reduced relative to the structure S_2_. We can notice that the absorption intensity exceeds 99% at peaks *P*_1_″ and *P*_2_″, and exceeds 95% at dip *D″*. As shown in Figure 3j–l, the electric fields can be better concentrated at the corner in S_3_, which demonstrates the collective oscillations of conductive electrons, evidently suggesting the existence of the LSPR mode [16]. In addition, the electric fields of S_3_ are much stronger than that of S_1_ and S_2_, proving that structure S_3_ can enhance the electric fields [36]. In this manner, structure S_3_ can also achieve broadband absorption based on the stronger localized electric field and low-Q LSPR.

**Figure 4:**
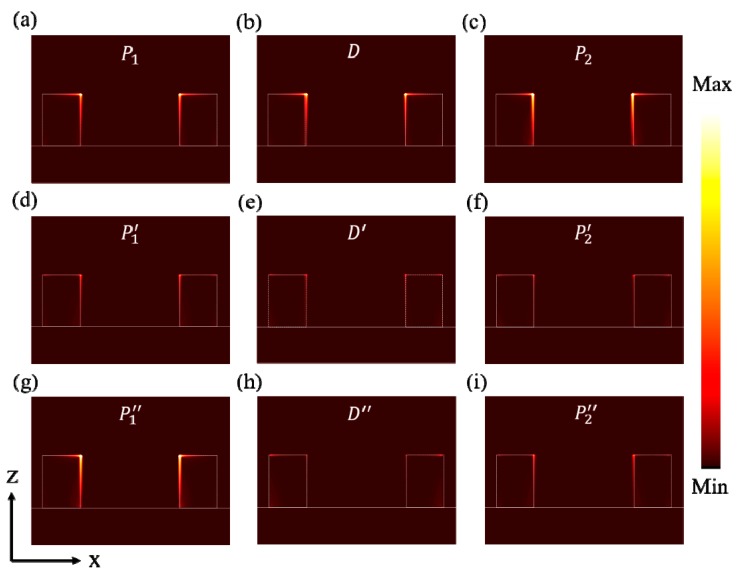
The cross-section colormaps describe the normalized power loss in the three structures. Structure S_1_ at (**a**) *P*_1_, (**b**) *D*, and (**c**) *P*_2_. Structure S_2_ at (**d**) *P*_1_’, (**e**) *D’*, and (**f**) *P*_2_’. Structure S_3_ at (**g**) *P*_1_″, (**h**) *D″,* and (**i**) *P*_2_″. Brighter areas correspond to higher power loss. The position of the cross-section is consistent with Figure 3.

To further and directly study the physical mechanism, we also studied the power loss distributions [19,23,39] of the three structures at the dip and two peaks. In this structure, the power loss comes from the resistance loss (*q*), which can be expressed by [40],

(1) $$q=\frac{1}{2}\varepsilon_{0}\omega \varepsilon_{m}^{\prime \prime }{\left|E\right|}^{2}$$

where *ε*_0_ is the vacuum dielectric constant, *ω* is the angular frequency of light, *ε_m_″* is the imaginary part of the relative permittivity of a metal (*ε_m_* = *ε_m_’* + *ε_m_″*), and *E* is the intensity of localized electric field. According to the Equation (1), the cross-section colormaps of the power loss are calculated and shown in Figure 4.

From profiles of the power loss in Figure 4, we can see that the power loss is mainly concentrated in Ti nanostructures. Power is absorbed at the corner and throughout inner edge region of the Ti nanostructures, which is consistent with the distributions of the electric field. By comparing Figure 3 and Figure 4, we can find that the variation trends of power loss intensity are in agreement with that of the absorption spectra from S_1_ to S_3_. In other words, the absorption magnitude of incident light is significantly enhanced when the structures change from S_1_ to S_3_. It supports the discussion that decreasing the short-side of the structure can enhance the localized electric field and result in the high absorption performance of Ti nanostructures.

**Figure 5:**
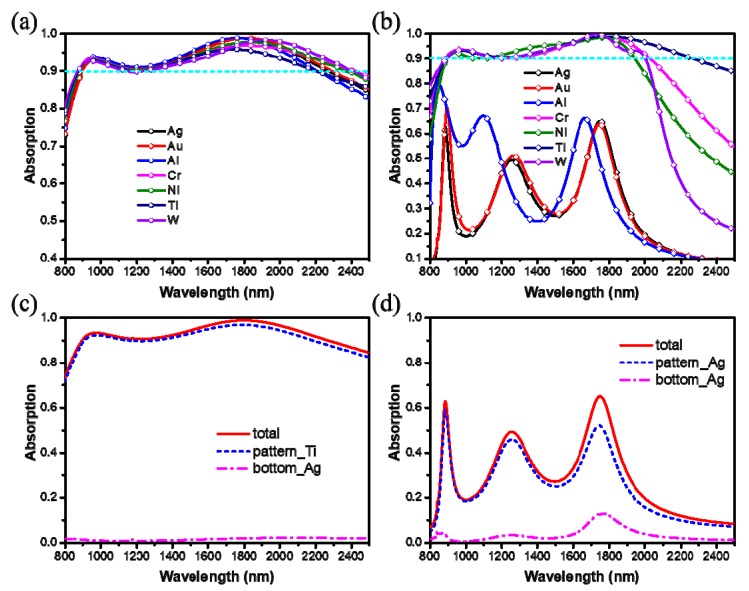
The contrastive absorption spectra of the broadband absorber when (**a**) metallic ground plane and (**b**) metallic nanostructures are replaced by the other metal materials. The contrastive absorption spectra of the total, pattern and bottom of (**c**) Ti-Ag structure and (**d**) Ag-Ag structure. Parameters: *Λ* = 800 nm, *L* = 700 nm, *w* = 150 nm, *h* = 280 nm, *t* = 100 nm, *l* = 275 nm.

We analyzed the effects of different types of composed metal material on absorption performance. First, Ti is used as the nanostructures, and Ag ground plane is replaced by metal materials (Au, Al, Cr, Ni, W, and Ti), the contrastive absorption spectra are shown in Figure 5a. We can see that only the absorption bandwidth changes slightly in this case. Next, using Ag as the ground plane and choosing metal materials (Ag, Au, Al, Cr, Ni, and W) to replace the Ti nanostructures, the contrastive absorption spectra are shown in Figure 5b. We can observe that using Cr, Ni, W, and Ti as the nanostructures, broadband absorption can be achieved and only the absorption bandwidth changes a little. Differently, using Ag, Au, and Al as the nanostructures cannot achieve broadband absorption, and there are only a few weak absorption peaks at the resonant frequency. In addition, using Ti and Ag as the nanostructures, we calculate the proportion of light absorbed by the surface metal pattern and the bottom metal film, as shown in Figure 5c,d. It indicates that the light is absorbed mainly by the surface metal pattern. Which is also consistent with the result of the power loss profiles shown in Figure 4.

**Figure 6:**
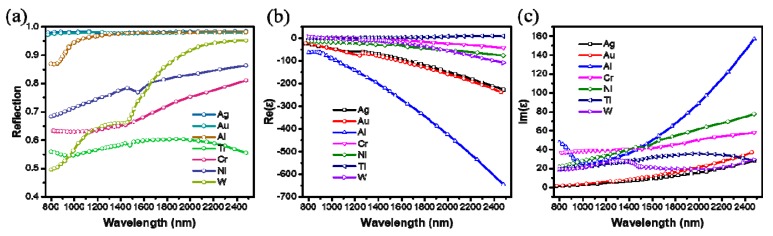
(**a**) The calculated reflection spectra of metal materials by the Equation (2). The real part (**b**) and imaginary part (**c**) of the permittivity of metal materials.

In order to clearly understand the primary cause why different types of metal materials lead to different absorption characteristics in the designed absorber, we use the Hagen-Rubens (H-R) relation [41] to describe the reflection and absorption performance of the metal film. The reflection (*R*) of metals at normal incidence can be derived by extended H-R relation [42,43],

(2) $$R=1-\frac{4n}{{\left(n+1\right)}^{2}+k^{2}}$$

where *n* and *k* are the real and imaginary parts of the refractive index of the metal. Figure 6a shows the calculated reflection spectra of the metal materials by the Equation (2). We can observe that most of the incident light is reflected (*k* is much more than *n*), and the rest is absorbed for the metals of Ag, Au, and Al. However, the reflection of the incident light reduces (*n* is close to *k*) for the metals of Cr, Ni, W, and Ti, which is to say they have intrinsic high absorption, especially in the short wavelength. In addition, the mechanism which the incident light is absorbed by the metal depends on that the Fermi level in metals locates inside a continuous energy band. Consequently, light that enters a metal can be absorbed with a tiny propagating length (skin depth), usually less than 100 nm [44]. Therefore, these metal materials used as the ground plane are opaque and have little effect on the absorption performance of the designed absorber when they are thicker than skin depth, which is consistent with the result shown in Figure 5a.

Accordingly, we can classify these metals into two types by their permittivity (*Re(ε) = n*^2^ − *k*^2^ and *Im(ε)* = 2*nk*), as shown in Figure 6b,c. First, Ag, Au, and Al are the first type of metals, which have high reflection property because of having large real part of the permittivity. This characteristic can lead to weak absorption because of impedance mismatch even if it has a large imaginary part of the permittivity, as in the case of Al. This also explains why broadband absorber cannot be achieved by using Ag, Au, and Al as the nanostructures, as shown in Figure 5b. Then, Cr, Ni, W, and Ti are the second type of metals, which is high loss metal materials because of having small real part and large imaginary part of the permittivity. Therefore, when they are used as the pattern layer of the absorber, it is easy to achieve impedance matching to decrease the reflection of the incident light. And strong localized electric field excited by the incident light, combining with high loss (high-*ε_m_″*) property of this metals can result in high resistance loss according to the Equation (1). Therefore, we can conclude that the high absorption of the designed absorber mainly comes from two parts. The first part is the power loss caused by the continuous electron transition excited by the incident light inside the metal. And the second part is the resistance loss mainly depends on the enhanced localized electric field caused by the nanostructures.

**Figure 7:**
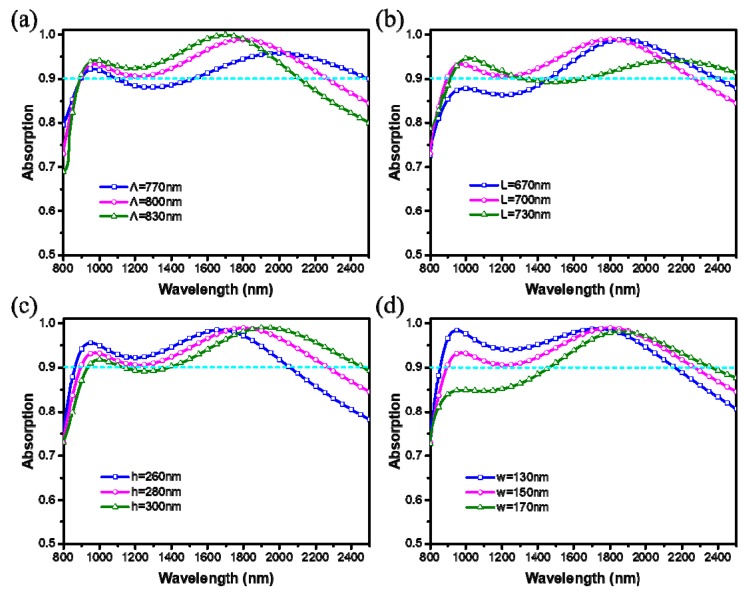
Simulated absorption spectra of the designed absorber under different geometric parameters: (**a**) period (*P*), (**b**) side length (*L*), (**c**) height (*h*), and (**d**) wire width (*w*) of the unit cell nanostructure.

In addition, we investigate numerically the absorption properties of the designed broadband absorber under different geometric parameters of the unit cell nanostructure. As shown in Figure 7, we present the absorption spectra with four different parameters (*Λ*, *L*, *h*, *w*), when changing only one parameter at a time, while keeping the other parameters unchanged. Similarly, through calculating the *RBW* (≥90%) of the curves, we find that the bandwidth of the absorber is first increased and then decreased with the increase of the geometric parameters. Differently, each parameter has a different effect on the change of the absorption curve. Figure 7a shows that the period (*Λ*) of unit cell may determine the long wavelength limit of *RBW* (≥90%). As *Λ* increases from 770 nm to 830 nm, the long wavelength limit is blue-shifted, the magnitude of the dip increase, resulting in *RBW* (≥90%) to increase and then decrease. From Figure 7b, although with the increase of the side length (*L*) the intensity of the short wavelength resonance peak is enhanced, but the magnitude of the dip will decrease to less than 0.9 when the *L* is bigger than 700 nm. Thus, the *RBW* (≥90%) is first increased and then decreased. Figure 7c shows the absorption efficiency as a function of the height (*h*). It indicates that as the *h* increases, the short and long wavelength limit and the position of two resonance peaks are red-shifted, but the intensity of the dip is reduced, which lead to first increase and then decrease of the *RBW* (≥90%). Finally, the wire width (*w*) has a more obvious effect on the absorption intensity at short wavelength. As the *w* increases, the long wavelength limit is red-shifted, but the absorption intensity at the short wavelength decreases rapidly, as shown in Figure 7d. The maximum *RBW* (≥90%) is found when the *w* is 150 nm. Therefore, the results of the bandwidth dependence on these parameters indicate that the optimized parameters are reasonable. In addition, the results of parametric analysis also indicate that the absorption properties can be tuned by varying the geometric parameters of the Ti unit cell nanostructure.

**Figure 8:**
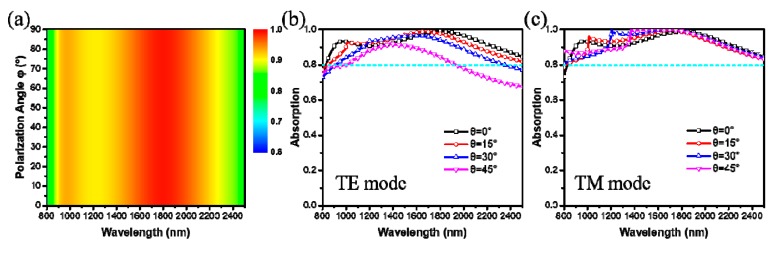
Simulated absorption as a function of (**a**) polarization angles *φ*; (**b**) and (**c**) incident angles *θ* with TE polarization mode and TM polarization mode. The parameters are the same as Figure 5.

For practical application, the absorption properties should consider the effect of polarization angle and incident angles. The effect of polarization angle on the absorption spectra has been studied at the normal incidence, as shown in Figure 8a. The absorption behavior does not show any variation with different polarization angles, which change from 0° to 90°. This is due to the high rotational symmetry of the unit cell nanostructure [38]. Furthermore, the absorption spectra of the absorber at oblique incidence have been calculated, the full-wave simulations are performed for both Transverse Electric (TE) polarization (the electric field of the incident light is parallel to *y*-axis) and Transverse Magnetic (TM) polarization (the magnetic field of the incident light is parallel to *y*-axis), as shown in Figure 8b,c. It is observed that obvious variation of the absorption spectrum happens at different oblique angles. In TE polarization, the absorption intensity drops obviously, and the *RBW* (≥90%) decreases dramatically with the increase of the incident angle. It is because the electric resonance mode cannot be excited effectively when the incident angle gets larger. In other words, electrics fields at large incident angles are confined less efficiently in the pattern layer than at normal incidence, hence leading to a weaker absorption. In TM polarization, one can clearly see the absorption intensity drops at the short wavelength with the increase of incident angle from 0° to 45°, which lead to a decrease of the *RBW* (≥90%). However, the decrease of the absorption for TM mode is not obvious compared with the one for the TE mode, since the magnetic response plays a dominant role over the electric response for TM mode. Therefore, the absorption intensity at short wavelength decreases slightly, and the absorption magnitude maintains higher value than 0.8 in the whole wavelength range. Thus, the proposed broadband absorber is polarization-independent at normal incidence and incident-angle-sensitive at oblique incidence. But for TM polarization, the absorption intensity can still be greater than 80% at an incident angle of 45°.

## 4. Conclusions

In summary, we proposed a broadband and polarization-independent absorber based on simple and all metallic metasurface working in the near-infrared region. The structure consists of metallic FITP array over a continuous Ag film. Under normal incidence, the simulative absorption is over 90% in the spectrum ranging from 895 nm to 2269 nm. Highly-efficient broadband absorption is ascribed to strong local effect of electric field and low-Q LSPR supported by Ti nanostructures. Specifically, through analyzing the electric field distributions, power loss profiles and reflection spectra of metal materials, we observe that the high absorption of the designed absorber mainly comes from the power loss caused by the continuous electron transition excited by the incident light inside the metal, and the resistance loss depends on the enhanced localized electric field caused by the FITP structure. For absorption spectrum, we can change the absorption bandwidth through tuning the geometric parameters of the nanostructures. Moreover, numerical simulations show that the broadband absorber is independent of the polarization angles at normal incidence, and has more than 80% high absorption persisting up to the incident angle of ~45° at TM polarization. Therefore, the broadband absorber may have potential applications in controllable thermal radiation and energy harvesting.
